# Cyclin-Dependent Kinase 4 is expected to be a therapeutic target for hepatocellular carcinoma metastasis using integrated bioinformatic analysis

**DOI:** 10.1080/21655979.2021.2006942

**Published:** 2021-12-12

**Authors:** Jia-Ning Zhang, Feng Wei, Lin-Han Lei, Yang Yang, Yuan Yang, Wei-Ping Zhou

**Affiliations:** a The Third Department of Hepatic Surgery, Eastern Hepatobiliary Surgery Hospital, Second Military Medical University, Shanghai, China; b Changzheng Hospital, Second Military Medical University, Shanghai, China; c Department of Hepatobiliary Surgery, Affiliated Hospital of Guizhou Medical University, Guiyang, China; d The Sixth Department of Hepatic Surgery, Eastern Hepatobiliary Surgery Hospital, Shanghai, China

**Keywords:** Bioinformatic analysis, CDK4, hepatocellular carcinoma (HCC), The Cancer Genome Atlas (TCGA), survival analysis, invasion and migration, epithelial mesenchymal transition (EMT)

## Abstract

Hepatocellular carcinoma (HCC) is the third leading cause of cancer-related mortality worldwide. HCC cells possess biological characteristics of high invasion and metastasis. In this respect, to prevent cancer cell invasion and metastasis and early active intervention, we herein screened through the TCGA database for further prognostic analysis including overall survival and disease-free survival . The Kaplan-Meier curve suggested that Cyclin-Dependent Kinase 4 (CDK4) might be an independent prognostic factor for HCC. Moreover, we performed mRNA expression analysis to measure CDK4 levels in normal liver tissues and HCC tissues, and immunohistochemistry analysis to detect protein level of CDK4 in Non-tumor tissue and HCC tissues . Our findings indicated that the expression of CDK4 was significantly higher in tumor tissues compared with Non-tumor tissue in HCC, which increased from HCC stage 1 to 3. Furthermore, the results of transwell-assay indicated that knocking down CDK4 significantly suppresses the invasion and migration of HCC cells, and the results of bioinformatics analysis revealed that genes closely associated with CDK4 are potentially worthy of further investigation. Additionally, the results of Western Blot indicated CDK4 regulates epithelial mesenchymal transition in HCC,and CDK4 appears to regulate EMT and HCC progression via the Wnt/β-catenin pathway. Collectively, this study found the key target gene through bioinformatic analysis and further functional validation through cell experiments. In particular, CDK4 is anticipated to become a crucial hub gene to snipe the metastasis of cancer cells in HCC.

**Abbreviations**: Hepatocellular carcinoma (HCC);Cyclin-Dependent Kinase 4(CDK4);Genomic Data Commons (GDC); genes; EC, Endometrial cancer; GEO, gene expression omnibus; GO, Gene Ontology; GSEA, Gene set enrichment analysis; KEGG, Database; TCGA, The Cancer Genome Atlas; TSGs, tumor suppressor genes;epithelial mesenchymal transition (EMT).

## 1 Introduction

Among gastrointestinal tumors, the incidence and mortality rate of liver cancer rank among the top five. Currently, surgical resection is the only curative treatment option for liver cancer [1,2]. However, when a patient develops distant metastasis of cancer cells and is subsequently classified as an advanced patient after TNM staging evaluation and cannot undergo surgery [3–5], then the survival benefit of such a patient is greatly reduced. Therefore, how to prevent the metastasis of liver cancer patients, as well as early detection and treatment are problems that require urgent attention.

With the recent wide application of RNA sequencing and the continuous supplementation and improvement of public biological databases, we can employ bioinformatics methods to identify key genes that can be used as a clinically applied tumor marker or a key target for treatment in The Cancer Genome Atlas (TCGA) [6–8] . In this study, our survival analysis and prognostic model screened out the immune gene CDK4 that was significantly associated with the survival and prognosis of liver cancer patients. Besides, it has been reported that CDK4 is highly expressed in liver cancer, and more often as a cell cycle regulator in cancer [9–12]. However, whether the up-regulation of CDK4 in liver cancer is related to the invasion and metastasis of cancer cells remains elusive and needs further investigation. Interestingly, we found that CDK4 was highly expressed in liver cancer database. Subsequently, we further confirmed the high expression of CDK4 in liver cancer samples. At the same time, we found that the expression level of CDK4 also changes during the period of cancer. Remarkably, we also identified that knocking down CDK4 can inhibit the invasion and migration in two HCC cell lines. In conclusion, this work aimed to screen and identify valuable genes and pathways via bioinformatic analysis and functional experiment validation of function.

Therefore, this study aimed at investigating the expression levels of CDK4 in HCC tissues, and we have also confirmed that CDK4 has great potential as a tumor marker to predict the prognosis of patients. Simultaneously,we also found the biological functions and definitive mechanisms of CDK4 in promoting HCC progression.

## 2 Materials and methods

### 2.1 Bioinformatic data collection

In this study, the transcriptome and clinical data of all LIHC in TCGA were obtained from Genomic Data Commons (GDC) Data Portal (https://gdc.cancer.gov/), whereby 373 clinical data were collected. Moreover, we obtained the immune gene data from the ImmPort data portal (https://www.immport.org/), where 1,604 immune-related genes were included. Then, all LIHC transcriptome data were analyzed for expression differences with the Wilcoxon test using R software, version 3.6.3. Univariate and multivariate Cox regression analyses were performed by the R package ‘survminer’ and ‘survival’. Heatmaps were made by the R package ‘pheatmap’. And raw data in our analyses is shown in (Supplementary material 1).We subsequently screened out the genes with significant expression differences between tumor tissues and normal tissues. The screening criteria were |log_2_FC| > 1 and FDR < 0.05. Finally, we used a Venn diagram to identify the differential immune genes from all differential and immune genes.

### 2.2 Validation of the hub gene

We analyzed follow-up data using the online software GEPIA (http://gepia.cancer-pku.cn/), to validate the differentially expressed genes (DEGs) from TCGA database. To further validate the selected oncogenes on the translational level, we used the ualcan online website (http://ualcan.path.uab.edu) to analyze both normal and cancer tissues.

### 2.3 Cell culture and treatment

For this experiment, we cultured human HCC cells (MHCC-LM3 and Sk-hep1) in Dulbecco’s modified Eagle’s media (Gibco, Shanghai, China), supplemented with 10% fetal bovine serum (FBS, Gibco, Shanghai, China) and 1% penicillin/streptomycin in a humidified incubator in an atmosphere of 5% CO_2_ at 37°C.

Additionally, the short hairpin RNA (shRNA) was synthesized and transfected into HCC cells using Lipofectamine 3000 (Invitrogen, Shanghai, China). Lastly, the targeting sequences for CDK4 control and shRNA are 5ʹ-AACTAACCAAGCGACAAGCTT and 5ʹ-GAGATTACTTTGCTGCCTTAA-3ʹ, respectively.

### 2.4 Immunohistochemistry (IHC)

In this subsection, IHC analysis was performed using human protein atlas database . The HCC tissue was obtained from the (Patient Id: 5031) whose pathology is liver cancer after surgery;the normal liver tissue was obtained (patient Id:2251) as comparison.

In addition, patients’ tissues were performed IHC analysis using rabbit polyclonal anti-human CDK4 antibodies (1:100, Abcam, USA) following the manufacturer’s instructions. we collected HCC tissues from patients diagnosed with hepatocellular carcinoma who underwent surgical resection at the Eastern Hepatobiliary Surgery Hospital, Second Military Medical University (Shanghai, China). We also obtained written informed consent from each patient, and the study was approved by our institutional review board of the Second Military Medical University.

### 2.5 Invasion and migration assays

Using transwell Matrigel invasive ability Chambers, we measured the ability of MHCC-LM3 and Sk-hep1 cells. First, the upper transwell chamber was coated with Matrigel (BD Bioscience, USA). Then, about 2 × 10^3^ cells were suspended in 200 uL serum-free media and added to the inserts. Afterward, the lower chamber was supplemented with 500 uL media containing 20% FBS and incubated at 37°C for 48 h before invasion assay. Moreover, cells that penetrated the upper layer were stained with crystal violet, and each experiment was repeated three times and the mean number of cells per field was recorded. Notably, the experimental procedure of the migration assay was roughly similar to that of the invasive assay except that the upper transwell chamber contained no Matrigel.

### 2.6 Western Blot

We extracted 20 μg of total protein from cultured cells, separated the proteins using 10% SDS-PAGE and transferred proteins onto polyvinylidene difluoride membranes. Then, we blocked proteins with 5% skim milk for 30 min and incubated them with diluted primary antibody (1:1000). Primary antibodies for GAPDH, CDK4, N-cadherin, Vimentin, Twist, E-cadherin, Wnt2, β-catenin, p-GSK3βand GSK3βwere purchased from Abcam (Cambridge, UK).

### 2.7 Statistical analysis

The results are expressed as mean ± SD. Differences between the two groups were compared using an unpaired Student’s t-test. Statistical data analysis was implemented with GraphPad Prism software 8.0 or SPSS software (IBM SPSS statistics, version 24). A level of *p* < 0.05 was deemed statistically significant.

## 3 Results

### 3.1 Identification of differentially expressed genes (DEGs) in HCC

**Figure 1.**
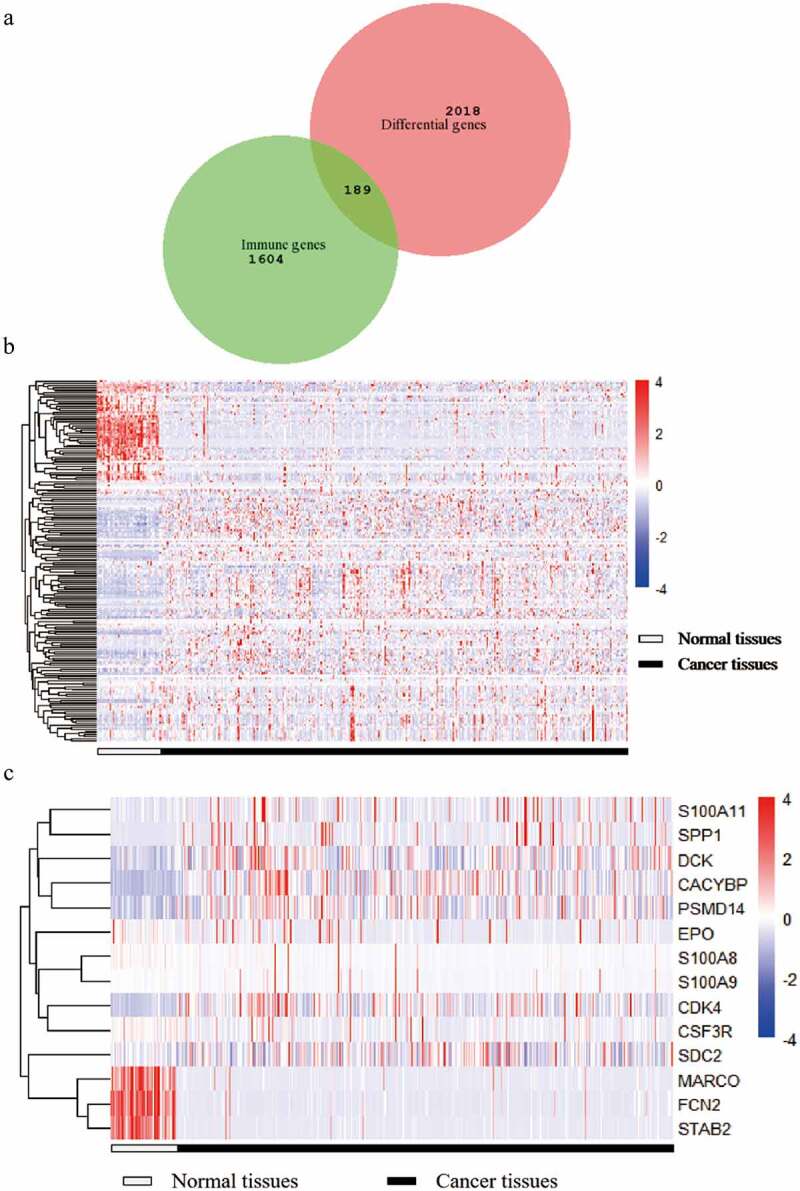
A Venn diagram and two heat maps of TCGA liver cancer. The Venn diagram (Figure A) shows the intersection of differential genes in TCGA liver cancer and immune genes, and 189 genes are obtained. The expression levels of differentially expressed immune genes in 89 liver cancers in normal and tumor tissues (Figure B). The expression levels of 14 target differential immune genes are screened out in normal and tumor tissues (Figure C)

After filtering the gene list using the Wilcoxon test, 2,018 differential genes met the criterion. Then, we obtained a total of 189 differential immune genes after all differential genes obtained were cross-screened with the immune genes (Figure 1a). The clustering of 189 differential immune genes was distinctly depicted in the heatmap between cancerous and adjacent tissues (Figure 1b). The p values and logFC values of the 189 differentially expressed genes is shown in (Supplementary material 2). Furthermore, univariate COX analysis of differential immune genes and clinical survival time and survival status yielded 14 differential immune genes related to prognosis: CDK4, CSF3R, CACYBP, DCK, EPO, FCN2, MARCO, PSMD14, S100A11, S100A8, S100A9, SDC2, SPP1, and STAB2 (Figure 1c, Table 1). Besides, multivariate COX analysis of differential immune genes show that 3 differential immune genes related to prognosis:CDK4,EPO,PSMD14.

**Table 1.** Univariate and multivariate analysis of differential immune genes related to prognosis

Gene	Univariate analysis		Multivariate analysis	
	P Value	Hazard Ratio	P Value	Hazard Ratio
CACYBP	0.005	1.000(1.000–1.001)	0.054	1.000(1.000–1.000)
CDK4	0.002	1.001(1.001–1.002)	0.001	1.001(1.001–1.002)
CSF3R	0.000	1.001(1.000–1.002)	0.69	1.000(1.000–1.001)
DCK	0.002	1.002(1.000–1.003)	0.226	1.000(1.000–1.000)
EPO	0.000	1.018(1.006–1.028)	0.03	1.001(1.000–1.001)
FCN2	0.006	1.009(1.003–1.012)	0.241	1.000(1.000–1.001)
MARCO	0.007	1.000(1.000–1.001)	0.784	1.000(1.000–1.000)
PSMD14	0.000	1.000(0.999–1.000)	0.023	1.000(1.000–1.001)
S100A11	0.002	1.000(1.000–1.001)	0.991	1.000(1.000–1.000)
S100A8	0.002	1.000(1.000–1.001)	0.812	1.000(1.000–1.000)
S100A9	0.000	1.003(1.001–1.004)	0.285	1.000(1.000–1.000)
SDC2	0.007	1.000(1.000–1.001)	0.283	1.000(1.000–1.000)
SPP1	0.007	1.007(1.002–1.015)	0.059	1.000(1.000–1.000)
STAB2	0.003	1.000(1.000–1.001)	0.326	1.000(1.000–1.001)

### 3.2 Hub gene screening of the 14 DEGs according to survival analysis

**Figure 2.**
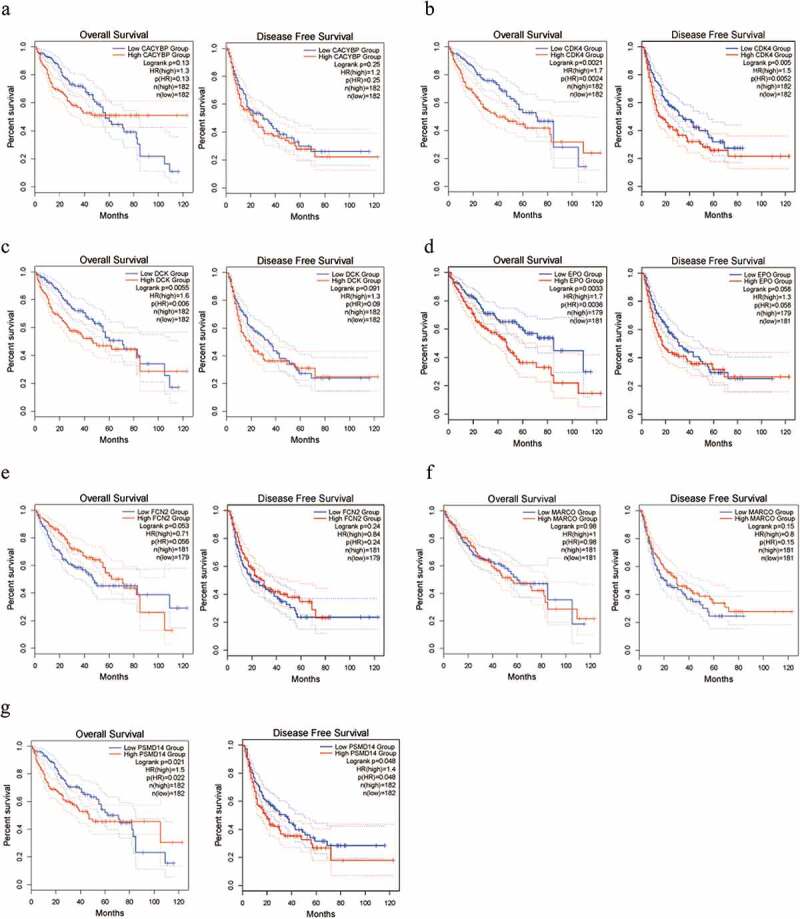
The survival analysis of the 7 hub genes performed using the Kaplan‐Meier curve. The expression of each gene showed its DFS and OS in HCC samples. The high expression of the hub genes CDK4 (Figure B) showed both OS and DFS are significantly poorer in HCC samples (Log-rank *p* < 0.05). Abbreviation: DFS, disease‐free survival; OS, overall survival; HCC, hepatocellular carcinoma

**Figure 3.**
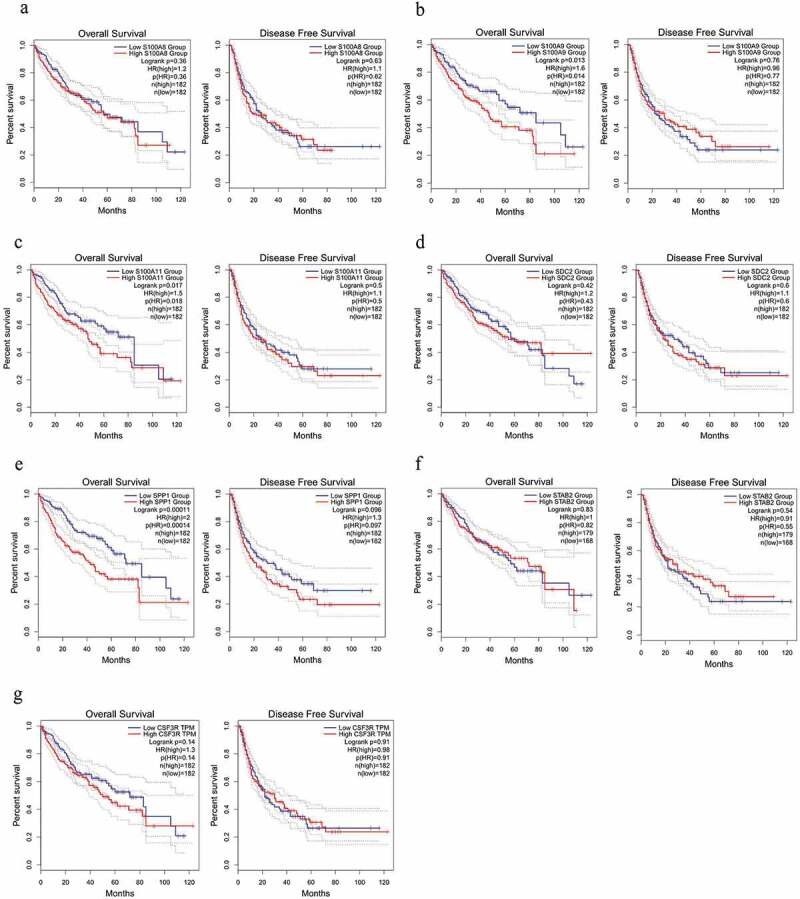
The survival analysis of the 7 hub genes performed using the Kaplan‐Meier curve. The expression of each gene showed its DFS and OS in HCC samples. Abbreviation: DFS, disease‐free survival; OS, overall survival; HCC, hepatocellular carcinoma

Based on prognostic analysis including overall survival and disease-free survival of the 14 DEGs that were screened through the GEPIA online website (Figure 2, Figure 3) and results (Figure 2b), we found that only CDK4 and PSMD14 showed significant statistical difference(all P values are less than 0.05) in both disease-free survival and overall survival of patients, whereas PSMD14 has been reported to be related to the proliferation and metastasis of hepatocellular carcinoma cells [13].Thus, the hub gene CDK4 is regarded as an important candidate gene for research.

### 3.3 Hub gene analysis

**Figure 4.**
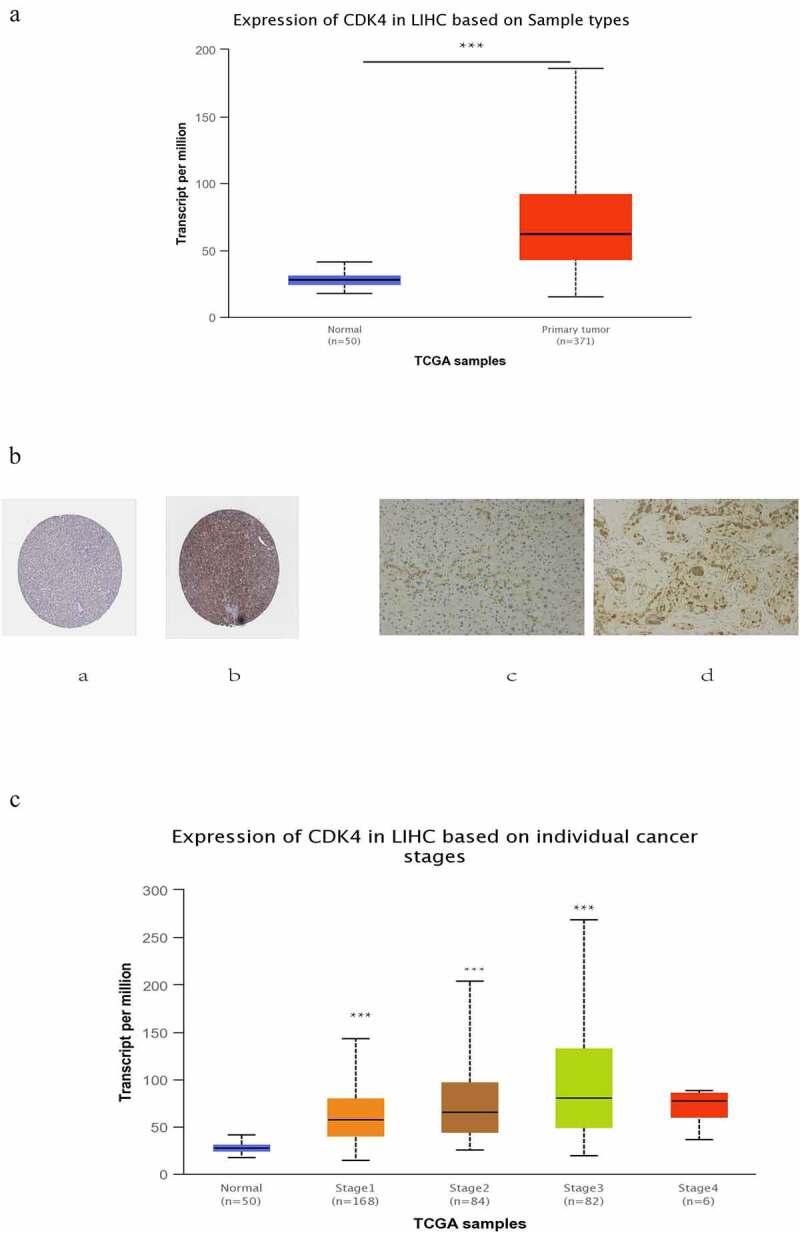
The expression of CDK4 in HCC tumor tissues and non-tumor tissue .(Figure 4 A) Transcriptional level of CDK4 expression was found to be in 371 HCC tissues compared with 50 normal tissues. Protein expression of CDK4 was significantly higher in HCC tissues(Figure 4b-b) compared with normal liver tissues(Figure 4b-a); and the Protein expression of CDK4 was also significantly higher in HCC tissues (^Figure^ ^4b-d)^ compared with paired paracancerous tissue tissues (Figure 4b-c) .(c) Transcriptional expression of CDK4 was significantly correlated with tumor grade, while patients in more advanced grade score expressed higher levels of CDK4 mRNA. The highest CDK4 mRNA expression was found in stage 3

After locking onto the CDK4 gene, we then analyzed the corresponding expression levels of CDK4 from the ualcan.path.uab.edu online website in both normal tissues and liver cancer tissues. We noted that the expression level of CDK4 was much higher in liver cancer tissues compared with that of normal tissues at the transcriptome level (Figure 4a). Similarly, the protein level of CDK4 was higher in tumor tissues than in nomal or paired paracancerous issues (Figure 4b), and its expression level of CDK4 was closely associated with the cancer stage (Figure 4c): Compared with the normal group,the later the tumor stage, the higher the expression level(due to too few cases in the stage4, there is no statistical difference. In general, in patients with liver cancer, the level of CDK4 expression was significantly correlated with advanced clinical-pathological parameters.

Furthermore, we make predictions on genes that are strongly related to CDK4 through the publicly available cancer OMICS data website (http://ualcan.path.uab.edu).The results showed that the gene SFRS9 was closely proportional to CDK4 (Supplementary Figure 1A), which possessed the highest Pearson Correlation Coefficient 0.83. Likewise, SFRS9 was also highly expressed in liver cancer (Supplementary Figure 1B), and its expression in cancer tissues was closely associated with the stage of cancer (Supplementary Figure S1 C). Furthermore, its high expression affected the survival of patients (Supplementary Figure 1D). Therefore, the function of CDK4 and its closely related genes in liver cancer is worthy of our further exploration.

### 3.4 In vitro *effects of CDK4 on HCC migration and invasion*

**Figure 5.**
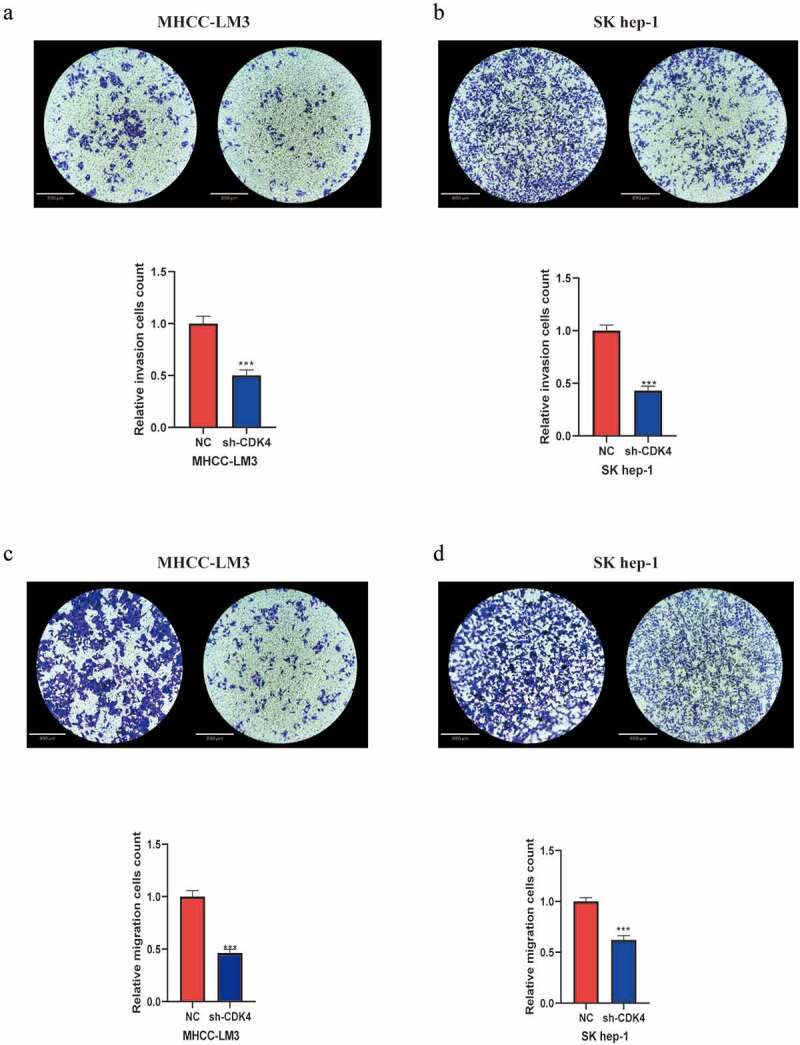
Knockdown of CDK4 attenuated HCC cell invasion and migration. (a) MHCC-LM3 cells transfected with sh-CDK4 were less invasive compared with control NC cells. (b) Sk-hep1 cells transfected with sh-CDK4 were less invasive as compared with control NC cells. (c) MHCC-LM3 cells transfected with sh-CDK4 were less migrating as compared with control NC cells. (d) Sk-hep1 cells transfected with sh-CDK4 were less migrating as compared to control NC cells. * = *p* < 0.05, ** = *p* < 0.01, *** = *p* < 0.001. Each independent experiment was replicated three times

After transfected and MHCC-LM3 and Sk-hep1 cells with shRNA specific for CDK4, we verified the transfection efficiency (Supplementary Figure 2), then explored the function of CDK4 on HCC migration and invasion. As shown in the invasion assay (Figure 5a and b), it was conducted to investigate the role of CDK4 in HCC cell lines. Our results revealed that knockdown of CDK4 can considerably impair cell invasion compared with control group cells. Next, we then performed the transwell migration assay to measure the role of CDK4 in the migration ability of HCC cells. As displayed in the migration assay (Figure 5c and d), we observed that knocking down CDK4 can markedly inhibit cell migration. Overall, these findings suggest that CDK4 may play an crucial role in affecting the invasion and migration of HCC cells, and hence it may be used as a treatment target to suppress cancer metastasis in the future.

### 3.5 CDK4 regulates epithelial mesenchymal transition and Wnt/β-catenin pathway in HCC cells

**Figure 6.**
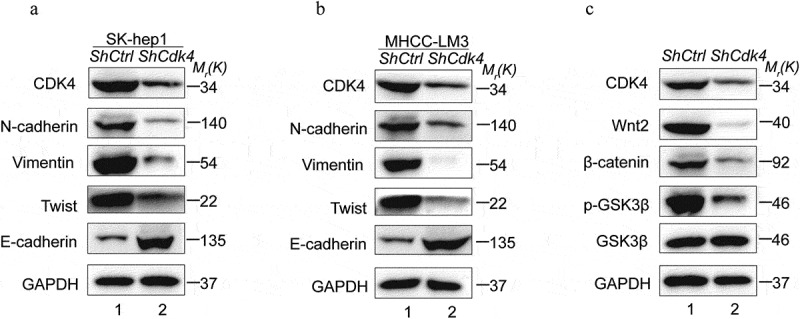
CDK4 regulates EMT markers and Wnt/β-catenin signaling pathway in HCC. (a) Western Blot analysis showed reduced N-cadherin, Vimentin, Twist and increased E-cadherin expression in Sh-CDK4 Sk-hep1 cells compared with normal controls. (b) Western Blot analysis showed reduced N-cadherin, Vimentin, Twist and increased E-cadherin expression in Sh-CDK4 MHCC-LM3 cells compared with normal controls. (c) Western Blot analysis showed reduced Wnt2, β-catenin, p-GSK3βexpression in Sh-CDK4 Sk-hep1 cells compared with normal controls, whereas the expression of GSK3βdidn’t change significantly

Epithelial mesenchymal transition (EMT) is an essential step for metastasis and correlates with poor prognosis in HCC patients. Thus, we detected the expression of EMT markers and analyzed the relationship between CDK4 and EMT in HCC cell lines. Western Blot analysis showed significantly increased E-cadherin and decreased N-cadherin, Vimentin and Twist protein levels in ShCDK4 cell lines compared with normal control cell lines, which performed both in Sk-hep1 and MHCC-LM3 cell lines (Figure 6a and b). The Wnt/β-catenin signaling pathway also plays a crucial role in regulating metastasis in HCC. Therefore, the key proteins of the Wnt/β-catenin pathway were be detected to observe the effect of CDK4 on this signaling pathway, such as Wnt2, β-catenin, p-GSK3β and GSK3β. The Sh-CDK4 Sk-hep1 cells showed lower expression levels of Wnt2, β-catenin, p-GSK3βcompared with the normal control cell lines, and with no significant change in GSK3β (Figure 6c). These results suggest that CDK4 regulates the Wnt/β-catenin pathway in HCC cells.

## 4 Discussion

To improve the overall survival time of HCC patients, effective prognostic markers and specific treatment strategies are very essential. There is no doubt that the metastasis of cancer cells is one of the big obstacles affecting the survival of HCC patients. In order to resolve these issues, we have carried out the exploration of this research. In this study, CDK4 expression in HCC tissues exceeded that in non-tumor tissues,and it showed the huge potential of being a tumor marker for predicting patients’ prognosis.Moreover, our findings indicated that CDK4 enhances tumor migration and invasion by modulating EMT via the Wnt/β-catenin pathway.All these findings highlighted CDK4 as an independent prognostic factor and a potential target for the treatment of hepatocellular carcinoma metastasis.

Among global cancer events, the morbidity and mortality of hepatocellular carcinoma still remain high [14–16]. One of the main reasons for this is that HCC cells are particularly prone to metastasis. For patients who have not undergone surgery, the distant metastasis of HCC cells will make the patient lose the opportunity for surgery and their survival prognosis will deteriorate. A current focus of cancer research is the development of agents that target specific oncogene in tumors [17]. Simultaneously,Recent insights into metastatic biology also reveal new potential targets that can be used for therapeutic advantage [18,19]. Therefore, we herein aimed to explore some genes or pathways with biological functions for early warning of HCC metastasis, or through active therapeutic intervention on corresponding molecular targets to prevent the further progress of cancer metastasis events.

In this study, we first crossed the immune-related genes through 2,018 differential genes in LIHC of the TCGA database, and then eventually selected CDK4 through survival and expression analyses on the ualcan.path.uab.edu online website. Specifically, CDK4, as a cell cycle regulatory protein, can play an essential regulatory role in the G1-S phase of cancer cells, thereby affecting the proliferation of cancer cells and promoting cancer progression [20,21]. Notably, the current CDK4 inhibitors such as ribociclib, abemaciclib, and palbociclib have been approved by the US FDA for the clinical treatment of cancer, achieving exciting results [10,22–25]. In this regard, we are primarily devoted to the related research of hepatocellular carcinoma. Previous studies on hepatocellular carcinoma have been reported that CDK4 exhibits a significant role in cell cycle regulation as a key factor in the signaling pathway, and thus can greatly inhibit the proliferation of cancer cells. As we mentioned earlier, the most important biological features of HCC are that it is prone to cancer cells’ invasion and metastasis. In this context, we therefore focused on whether CDK4 can affect the invasion and metastasis of cancer cells in HCC. We consecutively reviewed the previous literature, which revealed that there are very few related studies. After we found through the database that CDK4 was highly expressed in liver cancer as well as associated with the cancer stage, we subsequently explored it through a series of cell experiments. Our findings indicated that knockdown of CDK4 in the two HCC lines inhibited the migration and invasion of cancer cells, implying that CDK4 may play a critical biological function in HCC. At the same time, we found that CDK4 significantly regulates EMT in HCC cell lines, and is also subject to the Wnt/β-catenin pathway. Since CDK4 may be closely associated with the metastasis of liver cancer, future studies should thus mainly focus on the following two points for the corresponding clinical transformation. First, for patients after liver cancer surgery, whether CDK4 can be used as a tumor marker for related exploration, early warning of liver cancer metastasis, second liver cancer surgery, or early treatment for new metastatic lesions. In addition, based on website analysis, it predicted that the closely related gene of CDK4 was SFRS9, which was also highly expressed in liver cancer, and its expression was closely related to the stage of cancer. Therefore, these outcomes suggest that SFRS9 may also be a cancer-promoting factor in liver cancer,of course,whether it has a close relationship between SFRS9 with CDK4 and its related functions are worthy of further investigation. On the other hand, CDK4 may also be used as an effective therapeutic target key hub gene in the cancer pathway. Recently, it has been established that for HCC patients who have undergone metastasis, effective targeted drug therapy can be used to delay the progression of the disease or even cure it. Finally, it is important to note that CDK4 inhibitors have achieved good therapeutic effects in some other cancer studies. Likewise, our current experimental results show that CDK4 exhibits great potential as a therapeutic target in liver cancer cells, which warrant further exploration and validation.

## 5 Conclusion

In summary, this study demonstrates that CDK4 may participate in HCC development by modulating EMT through Wnt/β-catenin signaling and ultimately affect the survival of patients. Moreover, it may be used as a therapeutic target to fight against the migration and invasion of HCC cells.

## Supplementary Material

Supplemental Material

## Data Availability

All data in this study are available from both the GEO (www.ncbi.nlm.nih.gov/geo/) or TCGA(portal.gdc.cancer.gov) databases.
