# Supplementary material for: Cyclin-Dependent Kinase 4 is expected to be a therapeutic target for hepatocellular carcinoma metastasis using integrated bioinformatic analysis
Source: Bioengineered. 2021 Dec 12;12(2):11728–39. doi: 10.1080/21655979.2021.2006942 (PMC8810199; doi:10.1080/21655979.2021.2006942)
Supplement: Supplemental Material [file KBIE_A_2006942_SM1322.zip › supplementary/Supplementary Figure legend.docx]

Supplementary Figure 1. Finding the most closely related genes of CDK4 and its related functional analysis. (Supplementary Figure 1A) SFRS9 is the most closely related gene of CDK4. (Supplementary Figure 1B) The transcriptional level of SFRS9 expression was significantly higher in HCC tissues. (Supplementary Figure 1C) Transcriptional expression of SFRS9 was significantly correlated with tumor grade. (Supplementary Figure 1D) High expression of SFRS9 indicates a poor prognosis for HCC patients.

Supplementary Figure 2. The knockdown efficiency of CDK4 verification. The protein expression level of CDK4 in two HCC cell lines (MHCC-LM3 and SK Hep-1) were detected by by Western blots (A and B).
