# Supplementary figures and images for: Cyclin-Dependent Kinase 4 is expected to be a therapeutic target for hepatocellular carcinoma metastasis using integrated bioinformatic analysis
Source: Bioengineered. 2021 Dec 12;12(2):11728–39. doi: 10.1080/21655979.2021.2006942 (PMC8810199; doi:10.1080/21655979.2021.2006942)

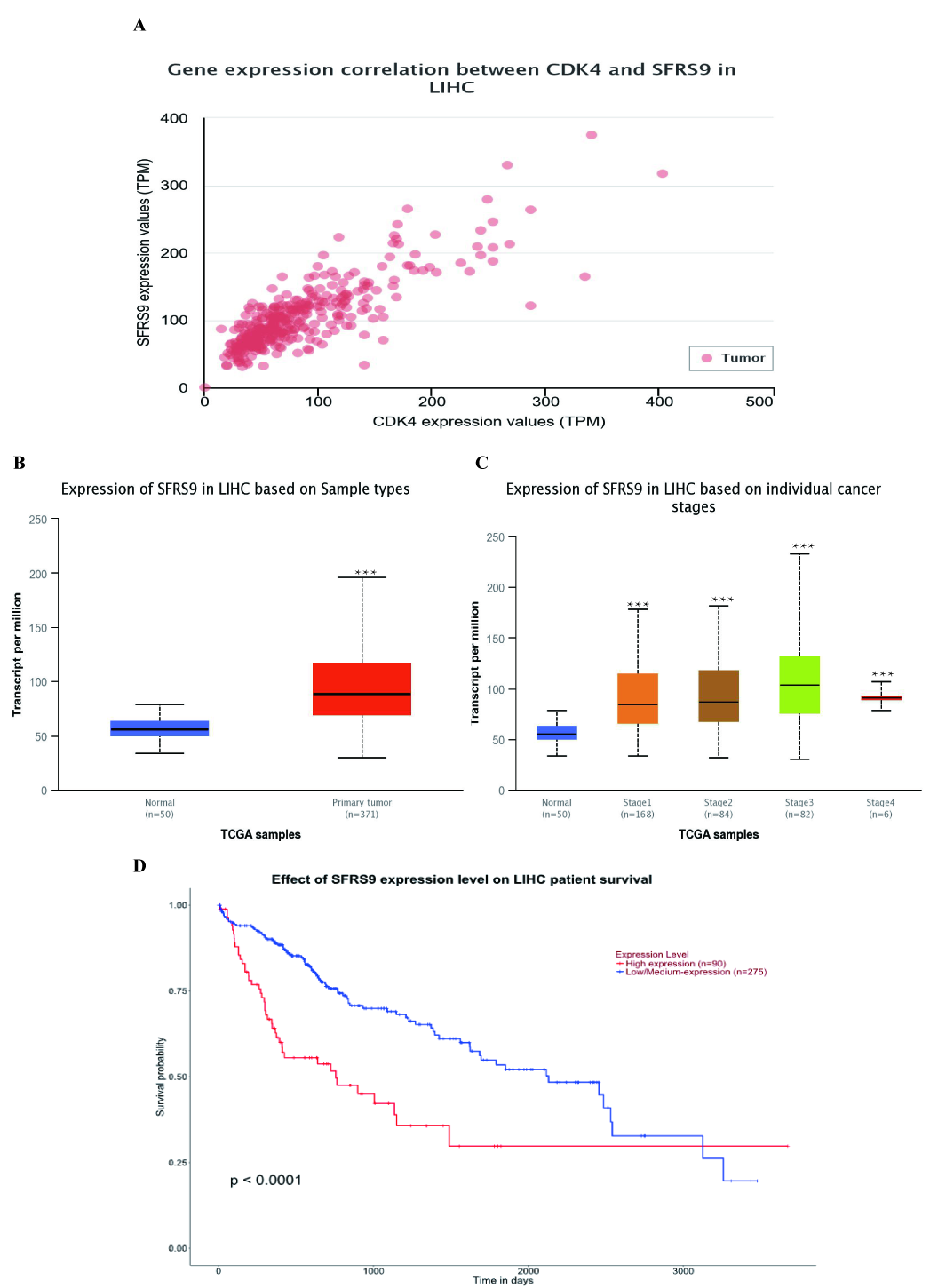

Supplement: Supplemental Material [file KBIE_A_2006942_SM1322.zip › supplementary/Supplementary Figure 1 (3).tif]

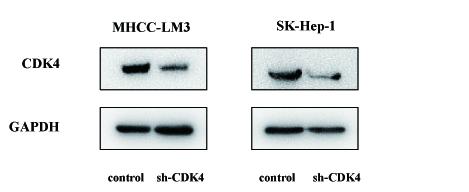

Supplement: Supplemental Material [file KBIE_A_2006942_SM1322.zip › supplementary/Supplementary Figure 2 (1).tif]
